# Intellectual abilities, language comprehension, speech, and motor function in children with spinal muscular atrophy type 1

**DOI:** 10.1186/s11689-021-09355-4

**Published:** 2021-02-02

**Authors:** Grazia Zappa, Antonella LoMauro, Giovanni Baranello, Emilia Cavallo, Priscilla Corti, Chiara Mastella, Maria Antonella Costantino

**Affiliations:** 1 SAPRE, Child and Adolescent Neuropsychiatric Service, Fondazione IRCCS Ca’ Granda Ospedale Maggiore Policlinico, Viale Ungheria 29, 20138 Milan, Italy; 2 Dipartimento di Elettronica, Informazione e Bioingegneria, Politecnico di Milano, Piazza Leonardo Da Vinci, Milan, Italy; 3 Dubowitz Neuromuscular Centre, NIHR Great Ormond Street Hospital Biomedical Research Centre, UCL Great Ormond Street Institute of Child Health, London, UK; 4 UO Neurologia dello Sviluppo, Fondazione IRCCS Istituto Neurologico Carlo Besta, Milan, Italy; 5 Child and Adolescent Neuropsychiatric Service, Fondazione IRCCS Ca’ Granda Ospedale Maggiore Policlinico, Milan, Italy

**Keywords:** SMA type 1, Language, Speech, Cognitive development, Children, Spinal muscular atrophy

## Abstract

**Background:**

Spinal muscular atrophy (SMA) is a chronic, neuromuscular disease characterized by degeneration of spinal cord motor neurons, resulting in progressive muscular atrophy and weakness. SMA1 is the most severe form characterized by significant bulbar, respiratory, and motor dysfunction. SMA1 prevents children from speaking a clearly understandable and fluent language, with their communication being mainly characterized by eye movements, guttural sounds, and anarthria (type 1a); severe dysarthria (type 1b); and nasal voice and dyslalia (type 1c).

The aim of this study was to analyze for the first time cognitive functions, language comprehension, and speech in natural history SMA1 children according to age and subtypes, to develop cognitive and language benchmarks that provide outcomes for the clinical medication trials that are changing SMA1 course/trajectory.

**Methods:**

This is a retrospective study including 22 children with SMA1 (10 affected by subtype 1a-1b: AB and 12 by 1c: C) aged 3–11 years in clinical stable condition with a coded way to communicate “yes” and “no”. Data from the following assessments have been retrieved from patient charts: one-dimensional Raven test (RCPM), to evaluate cognitive development (IQ); ALS Severity Score (ALSSS) to evaluate speech disturbances; Brown Bellugy modified for Italian standards (TCGB) to evaluate language comprehension; and Children’s Hospital of Philadelphia Infant Test of Neuromuscular Disorders (CHOP-INTEND) to assess motor functioning.

**Results:**

SMA 1AB and 1C children were similar in age, with the former characterized by lower CHOP-INTEND scores compared to the latter. All 22 children had collaborated to RCPM and their median IQ was 120 with no difference (*p* = 0.945) between AB and C. Global median score of the speech domain of the ALSSS was 5; however, it was 2 in AB children, being significantly lower than C (6.5, *p* < 0.001).

TCGB test had been completed by 13 children, with morphosyntactic comprehension being in the normal range (50). Although ALSSS did not correlate with both IQ and TCGB, it had a strong (*p* < 0.001) correlation with CHOP-INTEND described by an exponential rise to maximum.

**Conclusions:**

Although speech and motor function were severely compromised, children with SMA1 showed general intelligence and language comprehension in the normal range. Speech impairment was strictly related to global motor impairment.

## Background

Spinal muscular atrophy (SMA) is a genetic neuromuscular disorder, due to mutations in the survival of motor neuron 1 (SMN1) gene with an incidence of about 1 in 11,000 live births. It includes a wide range of phenotypes based on the age of symptoms’ onset and maximal motor achievement: very weak infants with prenatal/neonatal onset (SMA 0) or onset within 6 months of age and inability to sit unsupported (type 1, SMA1), nonambulant children able to sit independently (type 2), ambulant children (type 3), and adults (type 4) [1]. These maximal motor milestones, however, may be lost over time. Without any intervention, SMA1 is fatal in infancy [2, 3]. SMA type 1 can be subdivided into three groups with distinctly different natural histories. Type 1a is the severe neonatal variant, overlapping with the type 0, characterized by joint contractures with paucity of movement at birth and often needing immediate ventilatory support, therefore having a poor prognosis. Type 1b is the typical SMA1 form with patient having poor head control and difficulty in handling oral secretions upon or shortly after presentation and has an intermediate prognosis. Type 1c includes patients who can achieve head control or can sit with support and have the best prognosis, but it occurs in the minority of the cases. Higher copy numbers of the closely related *SMN2* gene is usually associated with milder clinical phenotypes [4, 5].

The progression of the disease is very rapid. Without ventilatory and nutritional support, death usually ensues within the first year of life because of respiratory failure [6–8]. The expectation of life has been extended with improved proactive nutritional and respiratory care, management of recurrent infections, development of orthopedics, and postural devices [9], so that some SMA1 patients began to survive into their teens.

In September 2017, AIFA (Agenzia Italiana per il Farmaco) authorized the first treatment for SMA in Italy. Nusinersen is an intrathecally administered antisense oligonucleotide designed to modify *SMN2* gene pre-mRNA thus increasing the level of SMN protein [10]. This has progressively changed the natural history of the disease, therefore showing new clinical phenotypes in treated patients. Knowing the natural history of the disease has become particularly important because benchmarks are needed to measure future treatment successes at different functional levels [11]. While the clinical phenotype and natural history of SMA1 is well known in terms of motor, respiratory, and bulbar/swallowing evolution [12–14], cognitive development and language comprehension of children and adolescents with this chronic disorder have not received much attention.

Communication has important implications in neurodevelopment, particularly for socialization, learning, and education, and it is strongly affected by the disease. Since the onset of symptoms, SMA1 has a severe impact on respiratory muscles [12] that, together with bulbar muscles, are the engine of speech function. Consequently, speech development is generally absent or very limited in SMA1 patients, with most of their communication being characterized by eye movements, guttural sounds, and anarthria (type 1a); severe dysarthria (type 1b); and nasal voice and dyslalia (type 1c) [14, 15]. Parents/caregivers usually try to translate the messages of the children or to replace them, with frequent communication breakdowns. This strongly limits the social interactions of SMA1 children only to the few persons able to understand their messages [16].

In clinical practice, there has been general consensus that cognitive function is well preserved in all forms of chronic SMA. However, studies on the development of cognitive and communicative abilities in children with SMA are still limited, particularly in the most severe type 1. Most of the published studies compared SMA types 2 and 3 with Duchenne muscular dystrophy [17], or relied on clinical/anecdotal reports on small groups of patients, or are outdated because based on antiquated methods not fulfilling current standards of child psychological research. For example, in 2002, Von Gontard et al. included 96 SMA children and adolescents, but they mostly belonged to the intermediate type 2 form. Eighteen children and adolescents defined as SMA1 were also tested and proved to have an average intelligence significantly higher than healthy controls. However, these eighteen patients seemed more likely to be borderline cases between 1 and 2, as the first signs of the disease were noticed at a mean age of 6.5 months [18]. SMA types 1a and 1b were probably not included in their study as the classification based on clinical subtypes was introduced only in 2005 [4]. In 1978, Hausmanowa-Petrusewicz used Binet and Wechsler scale to test IQ in SMA children and adolescents, concluding that SMA does not affect mental development [19]. A complete Wechsler test requires both verbal and performance parts, and therefore, it cannot be reliably administered to SMA 1a and 1b children. Most of the developed and standardized tests that explore neuropsychological constructs require not only language expressive skills, but also motor abilities, such as manipulating, handling small objects on command, building something, or using a pen to make signs on a page. These standardized formal assessments of cognitive functions are therefore strongly hampered by the severely compromised motor and speech abilities of SMA1 children. This may lead to underestimating their functioning. There is the need to adapt existing tests or to develop specific ones more appropriate for children with severe motor limitations [20]. For these reasons, only few data from natural history studies are available for SMA1 children and none of them referred to the most severely affected ones. In addition, nothing is reported about communication and cognitive development in the latest SMA standards of care recommendations [2, 3], particularly in SMA1.

In our service, since 2012, we developed a routine assessment that adapted existing standardized tests to the motor and speech limitations of children with SMA1 or with other rare diseases [21, 22], according to the existing literature [20, 23, 24]. Our goal was to monitor cognitive function, language comprehension, and quality of speech and optimize developmental interventions. Widely available standardized tests that could allow appropriate adaptation to the highly compromised motor functioning of patients were selected. In the standard procedure, the child has to point directly to the right picture, choosing between different alternatives. In the adapted scanning procedure, the examiners did manual scanning of the images in the tests to obtain the answers from the child, pointing with her finger to each of the different images, one by one, and asking the child to answer “yes” when the finger indicated the correct one or “no” when incorrect. All ways to communicate unequivocally “yes” and “no” were considered appropriate, including eyebrow movements, vocalisms, and specific eye movements. During the first part of the clinical evaluation session, the examiner was trained by parents to understand and familiarize with the child’s method of communication. In this way all the SMA1 children could complete both intellectual abilities and morphosyntactic comprehension tests. These adaptations were made to allow all SMA1 children accomplishing the tests, according to The Standards for Educational and Psychological Testing [25].

The routine assessment was short and administered in one session due to clinical severity/fatigability of the children, in order to fit in one of the periodic monitoring visits and to avoid multiple transfers to the family.

In order to bridge the gap between the clinical experience of high cognitive functioning in SMA 1 children and the lack of corresponding studies on this topic, we decided to retrospectively verify cognitive development, speech, and language comprehension in long-term natural history survivors [26]. We also aimed to verify whether and how these functions were influenced by patients’ age and by the severity of the disease.

## Methods

### Subjects and study design

This is a retrospective chart review study including all patients with clinical diagnosis and genetic confirmation of SMA 1 referred to a single Institution (Settore Abilitazione Precoce – SAPRE, Unita` Operativa Neuropsichiatria dell’Infanzia e dell’Adolescenza Fondazione IRCCS Ca` Granda Ospedale Maggiore Policlinico, Milan, Italy) between August 2012 and November 2017, and coming from all Italian regions. Only charts of children with the following criteria were included in the study: stable clinical conditions; 3 ≤ age ≤ 12 years; capacity to communicate “yes” and “no” by a coded way; not included in a clinical trial; and without (1) previous history of epilepsy, (2) severe visual impairment, (3) hypoxemia, (4) respiratory failure, (5) other clinical conditions requiring hospitalization one year before the tests, and (6) any other associated genetic disorder.

For all included charts, the following information were extracted: sex; reported age of symptoms onset; age at diagnosis; SMA1 subtype; ventilation; feeding method; head control; ability to sit or walk; wheelchair use; age at cognitive assessment; and results of cognitive, language comprehension, speech, and motor assessment.

The severity of clinical phenotype was classified on the basis of age at clinical symptoms onset and the ability to achieve head control into 1a, head control never achieved, signs at birth or in the neonatal period; 1b, head control never achieved, onset after neonatal period and by 3 months of age; and 1c, head control achieved, onset between 3 and 6 months of age [27]. The age of initiation of mechanical ventilation, either invasive (tracheostomy) or non-invasive, was also recorded.

G.Z. and P.C. reviewed the charts between September and October 2019. All patients had been assessed by the same operators during routine clinical practice, according to their specific functions: G.Z. administrated the cognitive and communication tests, C.M. evaluated the motor functioning, and P.C. collected the clinical data.

The Institutional review board has approved the chart review study (approval number: 0028609/11-07_2019bis).

### Tools

#### Intellectual abilities

Global intellectual abilities had been assessed by means of the Raven Coloured and Standard Progressive Matrices (RCPM), which is a one-dimensional test fulfilling the abovementioned requirements [28, 29]. The Raven’s CPM consists of 36 nonverbal items, distributed in three sets of 12 items (series A, Ab, and B); each item has six possible answers, being one correct and the other five incorrect. Item’s accuracy is dichotomized, where correct responses are scored with 1 point and wrong responses with 0 point, so that the maximum total score in each series is 12 and for the total scale is 36. RCPM raw score is the number of correct answers provided by the child and its percentile is then derived by comparing it to the normative Italian scores (average scores for age groups). The RCPM percentile is then converted into standardized IQ (70, 80, 90, 100, 110, 120, and 130) according to Raven et al. [30]. The test takes at least 45 min to be completed with the scanning procedure.

The Raven’s CPM has been used effectively in a wide variety of cross-cultural settings for children between the ages of 3.5 and 11. Children were asked to select a missing piece of a pattern out of six alternatives. Responses were marked in a paper protocol and later scored into correct and incorrect answers. Through this processing of visual information, the test is considered to be a measurement of ‘general intellectual abilities’ and the result can be expressed as IQ. The RCPM is somewhat unique as a general intelligence test. It focuses on visual problem solving, and in particular, on visual similarity and analogy. Using the RCPM as a measure of general intelligence, though it consists only of problems in a single, nonverbal format, stands in contrast to using broader tests like the Wechsler scales. These scales include subtests across several different verbal and nonverbal domains. RCPM has been used frequently in studies of children with speech and language impairment [31] because it is non-verbal, comparatively short to administer and engaging [24]. These features make it a suitable measure of mental age in children, who often have limited language comprehension and expression.

An Italian standardization for children aged 3.5 to 11 years is available [30].

#### Morphosyntactic comprehension

Morphosyntactic/syntactic comprehension had been assessed by means of the Test of Grammatical Comprehension for Children (Test di Comprensione Grammaticale per Bambini, TCGB) [32]. Children had to choose pictures corresponding to target sentences uttered by the examiner, discriminating them among morphological-morphosyntactical distracters. In this test, each item has been designed to tap a specific kind of sentence (declarative, relative, negative, passive, etc.), with 4 pictures between which to choose. TCGB raw score is the number of wrong answers provided by the child and its percentile is then derived by comparing it with standard score curves. The normal range of variation is considered between 90th (upper limit) and 10th (lower limit) percentiles. Values between the 25th and the 10th percentile are to be interpreted as borderline, especially for the lower age groups [32].

The test assesses morphosyntactic comprehension and is the Italian adaptation of the Brown Bellugi test. It can be administered in children from 3.5 years. The test takes at least 90 min to be completed with scanning procedure.

#### Speech

The level of speech disturbances had been assessed by means of the ALS Severity Score (ALSSS) developed to evaluate patients with amyotrophic lateral sclerosis (ALS) [33]. The score can provide a rapid and accurate assessment of the patient’s disease status when combined with measurement of vital capacity in patients with motor neuron disease. It includes four domains: (1) speech, (2) swallowing, (3) lower extremity and walking, and (4) upper extremity dressing and hygiene. As ALSSS is not specific for SMA patients, we considered only the speech domain for our study. This is a 10-point scale ranging from 1 (non-vocal) to 10 (normal speech processes).

#### Motor function

Motor skills had been assessed by means of the Children’s Hospital of Philadelphia Infant Test of Neuromuscular Disorders (“CHOP INTEND”). The CHOP INTEND is a reliable, easily administered, and well-tolerated motor test for SMA1 and similarly weak infants with neuromuscular diseases. The CHOP INTEND can provide a useful measure of motor skill in this population both for clinical monitoring and for research studies, and it is currently used as outcome measure in all the ongoing clinical trials. It includes 16 items with a total score ranging from 0 to 64, with higher scores indicating higher abilities. The scale was performed by a clinical evaluator properly trained. The CHOP INTEND can be done in a short period of time and does not place infants in positions that are poorly tolerated. This instrument allows examining strength during reflexive, spontaneous, or goal-directed movement, while also examining the behavioral state of infants. CHOP INTEND covers a very large age range (3 months to 21 years) [34].

### Statistical analysis

Motor function, cognitive abilities, speech quality, and language comprehension from the retrospective chart analysis were firstly tested with Kolmogorov-Smirnov normality test. In order to verify if they differed according to age or phenotypes, a one-way analysis of variance or Kruskal-Wallis one-way analysis of variance on ranks were used with age or clinical phenotypes of SMA1 as independent factor, respectively if the variable did or did not pass the normality test. A Spearman rank order correlation analysis was performed to explore the relationship among the considered variables and the Spearman correlation coefficient (*ρ*) and the corresponding *p* value were computed. In case of significant correlation (i.e., high *ρ* and *p* < 0.05), the best (highest regression coefficient, *R*) among linear, polynomial, and exponential regression analysis was chosen (SigmaStat version 11.0; Systat Software, San Jose, CA, USA).

Significance was set as *p* < 0.05. Data in the “Results” section of the text are reported as median value.

## Results

### Clinical characteristics of the sample

Between August 2012 and November 2017, seventy-four patients with a diagnosis of SMA1 accessed our service, all their charts were revised, and thirty-five fulfilled the inclusion criteria. Among these, the data of three children could not be used because their parents had not signed informed consent for clinical data collection, and ten charts were excluded due to incomplete data collection. Charts from the remaining twenty-two patients (8 males, 14 females) aged between 3 and 11 years (Table 1) were reviewed for this study.

**Table 1:** Demographic and personal characteristics of the 22 SMA1 children

Sex (%)	
Male	36
Female	64
**Age (years)**	
Median	5
Range	3–11
**SMA1 subtype (%)**	
1a	9
1b	36
1c	55
**Age at diagnosis (months)**	
Median	6.2
Range	0.1–9
**Mechanical ventilation (%)**	
Invasive ventilation	31.8
Non-invasive ventilation	68.2

**Fig. 1:**
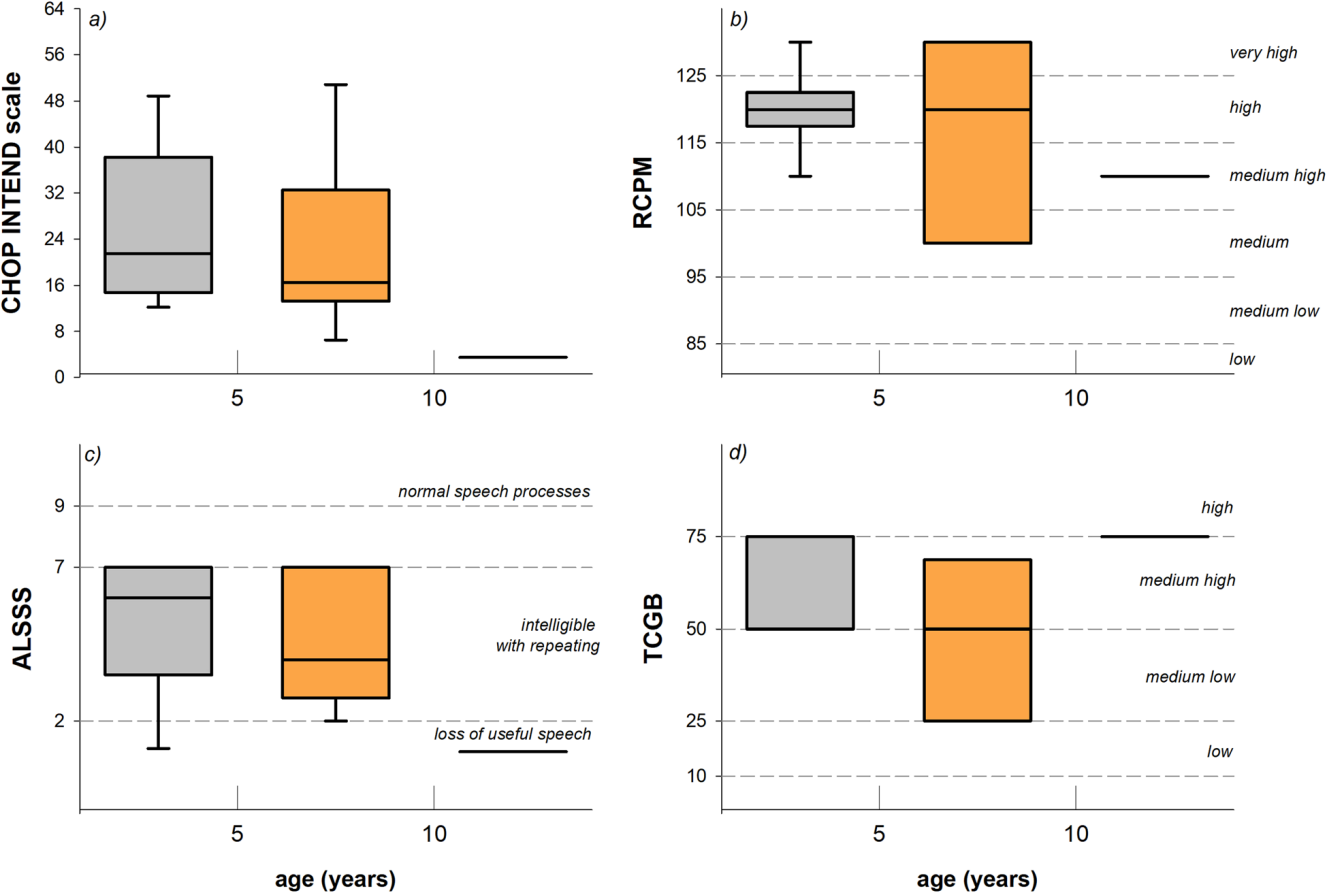
Box-and-whisker plot representing the median (line within the box), the interquartile range (length of the box), the 90th and the 10th percentiles (whiskers above and below the box) of Children’s Hospital of Philadelphia Infant Test of Neuromuscular Disorders (CHOP INTEND, **a**), intellectual abilities (RCPM, **b**), quality of speech (ALSSS, **c**), and language comprehension (TCGB, **d**) in children whose age was < 5 years (grey); ≥ 5 and < 10 years (orange); and ≥ 10 years (black). The horizontal short-dashed lines indicate the different levels of intellectual abilities, quality of speech, and language comprehension

The 22 eligible patients were firstly split in three groups according to age, and therefore to disease progression: ten children whose age was < 5years (5 SMA 1b and 5 SMA 1c); ten children whose age was ≥ 5 and < 10 years (2 SMA 1a, 1 SMA 1b and 7 SMA 1c); and two children whose age was ≥ 10 years (both SMA 1b). CHOP INTEND score was similar between the groups (*p* = 0.056), although it was severely lower in the two oldest patients (Table 2 and Fig. 1a).

**Table 2:** Age and clinical data of SMA1 children

Patient	Gender	Data test	SMA1 type	Age (yrs)	ALSSS (/10)	RCPM (IQ)	RCPM (percentile)	TCGB (percentile)	CHOP INTEND (/64)
#1	F	August 2012	1B	5.0	2	130	100	75	6
#2	F	June 2012	1B	7.0	4	120	92	50	15
#3	F	October 2012	1B	6.1	3	110	73	25	11
#4	F	January 2013	1B	4.0	4	120	89		12
#5	F	February 2013	1B	11.0	1	110	67		4
#6	M	March 2013	1B	10.0	1	110	82		3
#7	M	September 2013	1A	3.1	2	120	90		17
#8	F	May 2016	1B	5.1	4	130	98	75	15
#9	M	November 2016	1A	4.0	1	110	67		15
#10	M	April 2017	1B	5.0	2	120	93	75	14
#11	F	May 2012	1C	4.1	6	110	74	50	38
#12	F	August 2012	1C	6.1	6	120	89	25	18
#13	F	October 2012	1C	5.1	4	100	48	50	18
#14	M	January 2013	1C	4.1	6	120	93		14
#15	F	January 2013	1C	7.1	7	130	96		49
#16	F	June 2013	1C	9.1	7	100	61	25	51
#17	F	February 2014	1C	3.1	7	120	89		50
#18	F	June 2014	1C	4.1	6	120	92		39
#19	F	May 2016	1C	4.0	7	130	98	50	20
#20	M	May 2016	1C	5.0	7	100	56	50	27
#21	M	March 2017	1C	4.1	6	130	98	75	23
#22	F	November 2017	1C	3.1	7	120	84		37
		**SMA1 subtypes A-B**	**Median**	**5.0**	**2**	**120**		**75**	**13**
			**25th perc**	**4.3**	**1**	**110**		**50**	**7**
			**75th perc**	**6.8**	**4**	**120**		**75**	**15**
		**SMA1 subtype C**	**Median**	**4.1**	**7**	**120**		**50**	**32**
			**25th perc**	**4.1**	**6**	**108**		**38**	**20**
			**75th perc**	**5.3**	**7**	**123**		**50**	**42**

*F* female, *M* male, *ALSSS* ALS Severity Score, *RCPM* Raven Coloured and Standard Progressive Matrices, *TCGB* Test di Comprensione Grammaticale per Bambini (Test of Grammatical Comprehension for Children), *CHOP INTEND* Children’s Hospital of Philadelphia Infant Test of Neuromuscular Disorders, *25th perc* 25th percentile, *75th perc* 75th percentile

The 22 eligible patients were also split in two groups according to the severity of the disease: ten children affected by the most severe forms (AB: 2 SMA1a and 8 SMA1b), and twelve SMA1c (C). As shown in Table 2, AB and C children were similar in age (respectively 5.0 and 4.1 years, *p* = 0.409); however, the former were characterized by lower CHOP INTEND scores compared to the latter (respectively 13 and 32, *p* < 0.001).

The analysis of the charts revealed that seven patients (31.8%, all belonging to AB) were on tracheostomy, and 15 patients (68.2%) were on non-invasive ventilation. Twenty patients were fed by gastrostomy. First signs of the disease were noticed at a mean age of 4.4 months and diagnosis at 6.2 months. None of the patients achieved the ability to sit unsupported and/or to walk independently. Fifteen children used powered wheelchair, seven with scanning and micro-light and eight with mini-joystick. All patients needed a sitting aid. Nine patients (40%) were recorded to remain reclined and seven (33%) to need mechanical ventilation during the cognitive assessment. With one exception, all the children were reported to regularly attended schools or kindergartens, with home school programs during wintertime or when they were sick. Eighteen children were Italian, four were exposed at home to a different language either because of their family being recently relocated to Italy, or because living in a bilingual region of Italy.

All children had received home physiotherapy and fourteen also had a speech language rehabilitative intervention once a week. All the families had been trained to the use of augmentative alternative communication systems; however, eight had not used them in their child daily life. Because we decided to review charts only until 2017, no child was receiving any approved or investigational SMN-restoring treatment for SMA.

### Intellectual abilities, speech, and morphosyntactic comprehension

Both RCPM and ALSSS tests were reported in the charts of all 22 children (Table 2).

**Fig. 2:**
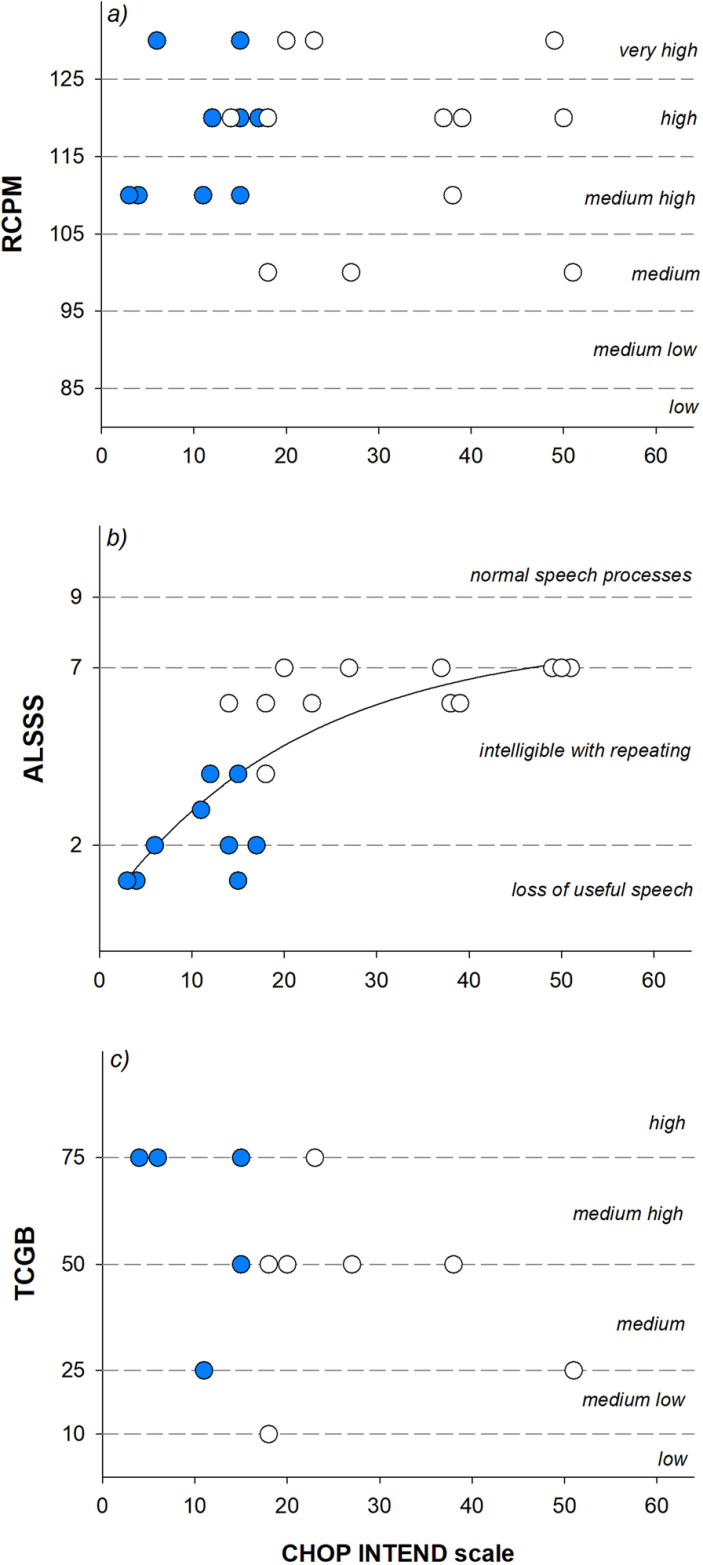
Correlation between Children’s Hospital of Philadelphia Infant Test of Neuromuscular Disorders (CHOP INTEND, *x-axis*) and intellectual abilities (RCPM, **a**), quality of speech (ALSSS, **b**), and language comprehension (TCGB, **c**) in children affected by the most severe SMA1 a and b forms (blue symbols) and by c form (white symbols). The horizontal short-dashed lines indicate the different levels of intellectual abilities, quality of speech, and language comprehension

The median IQ of the overall population was in the high level (120) ranging from 100 to 130, (Table 2, Fig. 1b). There was no significant difference between pre-school (age < 5 years) and school children (age ≥ 5 years, *p* = 0.331) and also between AB and C (*p* = 0.945, Fig. 2a).

Global median score of the speech domain of the ALSSS was 5, ranging from 1 (non-vocal) to 7 (detectable speech disturbances with obvious speech abnormalities), with no difference between children younger than 5 years and children whose age ranged between 5 and 10 (*p* = 0.587). Due to tracheostomy, vocal function was severely compromised in the two oldest patients (*p* = 0.015), as shown in Fig. 1c. However, when considering the clinical phenotypes (Table 2 and Fig. 2b), ALSSS was significantly lower in AB children, compared to C (*p* < 0.001).

TCGB test was reported in the charts of thirteen children (Table 2). The test had not been administrated to the four children exposed at home to a different language, whereas the five remaining children did not complete the assessment due to either fatigue or lack of attention. The global median TCGB score was 50 (Fig. 1d), being similar among the three groups of age (*p* = 0.392), while it tended to be higher in AB than C children although not significantly (*p* = 0.268).

### Correlations

**Fig. 3:**
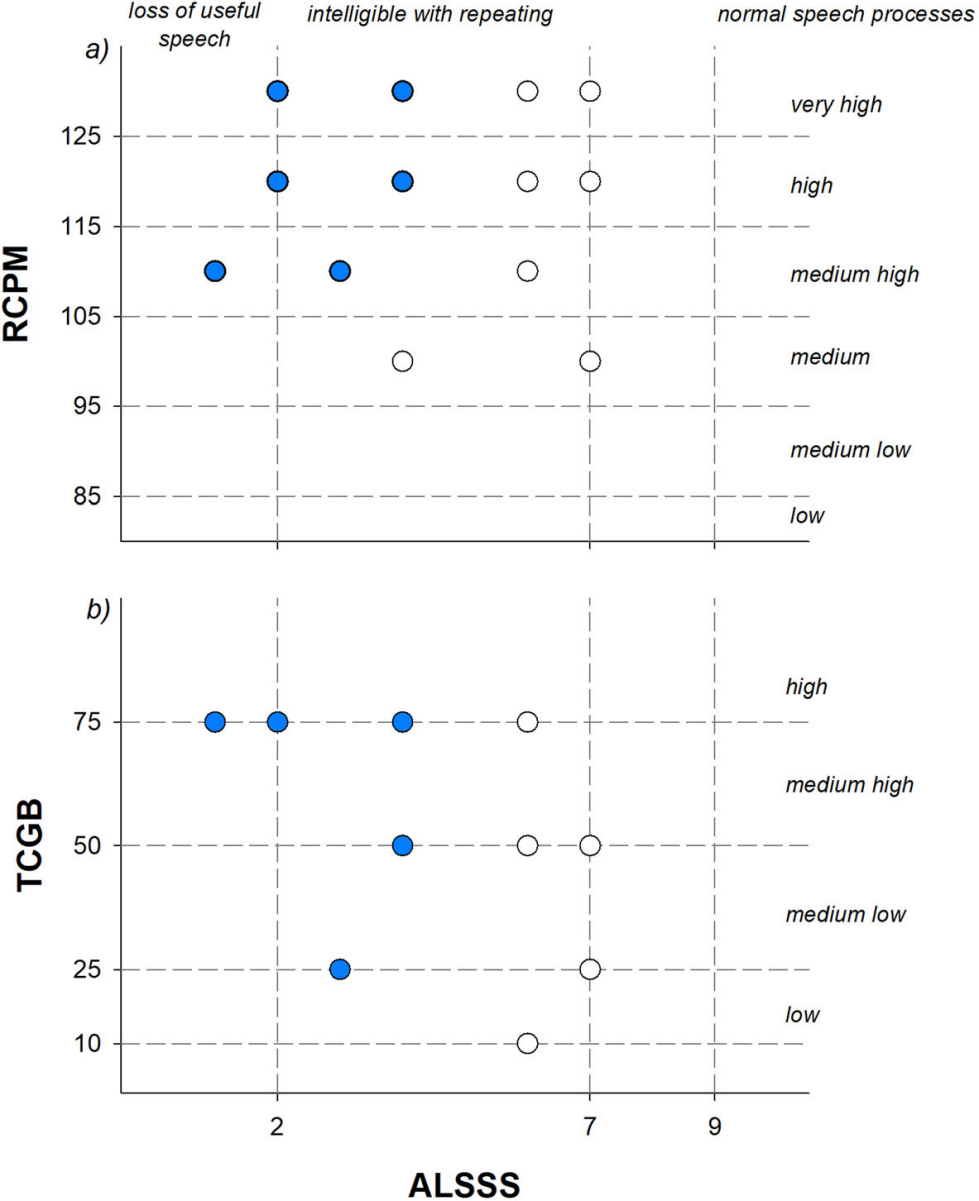
Correlation between quality of speech (ALSSS, *x-axis*) and intellectual abilities (RCPM, **a**) and language comprehension (TCGB, **b**) in children affected by the most severe SMA1 a and b forms (blue symbols) and by c form (white symbols). The short-dashed lines indicate the levels of intellectual abilities and language comprehension and the threshold of speech disturbances. The number points seemed lower because some children have the same RCPM and TCGB scores; therefore, some points are overlapped

Figure 2 shows the correlations between motor functional scale and intellectual abilities, speech, and language comprehension. A strong correlation (*ρ* = 0.833; *p* < 0.001) was found between CHOP INTEND and ALSSS, whereas there was no correlation with IQ (*ρ* = 0.0154; *p* = 0.944) or TCGB (*ρ* = − 0.370; *p* = 0.224). ALSSS did not correlate with both IQ (*ρ* = 0.157; *p* = 0.482) and TCGB (*ρ* = 0.285; *p* = 0.098), as shown in Fig. 3. The best fitting curve (*R* = 0.83; *p* < 0.001) describing the relationship between CHOP INTEND and ALSSS was an exponential rise to maximum: *y* = *a* (1-exp ^(−*bx*)^); where *y* was ALSSS and *x* CHOP INTEND scale. The two parameters, *a* and *b*, were respectively 7.88 and 0.047, obtained after 7 iterations.

## Discussion

To our knowledge, this is the largest study investigating cognitive abilities, language comprehension, and speech in untreated children with SMA type 1, and their relation with motor capacities. It is the very first one considering also the different phenotypes of SMA1, including the most severe forms of the disease. Cognitive function and morphosyntactic comprehension are well preserved in all SMA1 children, while speech, like motor function, is strongly affected by the severity of the forms. Phenotypes, rather than age, seemed to be the most discriminant factor that influences the quality of expressive language in SMA1 children.

Our routine model of assessment had been introduced to overcome the difficulties encountered by clinicians in the evaluation of cognitive functioning and communication in SMA1 in everyday clinical practice. Due to severe degree of neurologic dysfunction and severe dysarthria in the majority of SMA1 patients, only tests requiring neither motor nor verbal components nor set time-limits are appropriate to be used. However, they need to be adaptable to visual scanning. Without interpretable means of expression, assessment of cognitive skills in SMA1 is an imposing task that heavily relies on performance [16]. Adaptations of the original test or the administration process can help increase access for some individuals and they are typically classified as accommodations or modifications. The Standards for Educational and Psychological Testing [25] clarifies that accommodations involve changes in test administration without changing the underlying construct measured by the instrument, thereby retaining the comparability of scores.

We evaluated speech quality by using the ALSSS speech scales developed for ALS, being the most phenotypically similar disease to SMA1. Although this tool was developed for adult patients, it was easily applicable to SMA1 children because it allows to evaluate patients with severe dysarthria including non-vocal processes.

A dynamic system perspective suggests that motor function and speech development are intimately linked during infancy and toddlerhood [23]. This was confirmed by our results, since ALSSS resulted to be strongly dependent on the SMA1 phenotypes, and on CHOP INTEND scale. The relation that we found was described by an exponential rise resulting into a plateau around the score of 7 (Fig. 2b). This means that the natural history of SMA1 is characterized by speech disturbances even at highest CHOP scale values. Age did not seem to play a role in ALSSS, with the exception of the two elderly patients, aged 10 and 11 years, who were both tracheotomized and without functional speech.

The only applicable test to quantify general IQ, suitable for the severely limited motor ability and lack of speech of SMA1 children, was RCPM. When compared to other scales, RCPM was shown to provide more comprehensive information on cognitive performance on adult cerebral palsy patients [35] and on children affected by cerebral palsy or other motor and speech disorders, however not as compromised as in SMA1 a and b [28, 29].

SMA1 children IQ scored in the high range, regardless of age, disease severity, and phenotypes, being in agreement with the only two authors that studied cognitive functions in SMA1. One of these, however, was a preliminary study on four children [36], while the other included a small subgroup of SMA type 1 children, being mostly borderline between types 1 and 2 [18].

RCPM evaluates only the fluid component of the intelligence; for this reason, an adapted TCGB had been chosen to investigate also language morphosyntactic comprehension, particularly in non-verbal children. In general, SMA1 children were characterized by medium-high levels of morphosyntactic comprehension irrespective of age, motor function, and disease subtypes. This implies that the cognitive and morphosyntactic comprehension functions are well preserved or even increased as also suggested by the clinical experience.

For this reason, we have not found correlation between ALSSS, RCPM, and TCGB, while the only important correlation was between ALSSS and CHOP INTEND scale. These results confirm that young SMA1 children with restricted locomotive activity and limited manipulatory experiences are not affected in the establishment of intellectual potential and in the development of effective comprehension skills, while speech is severely compromised.

However, the progressive lack of stimulation and the limited social experiences in older SMA1 children could lead to a gap between cognitive abilities and grammar comprehension. The former might remain stable, while we expect the latter to get worse, being strongly dependent on exposure to language, book reading, and learning programs.

Finally, we demonstrated that the severe speech impairment induced by the disease does not seem to affect both the intellectual abilities and language understanding (Fig. 3).

Our results have important consequences in the global management of SMA1 children. Indeed, cognitive ability and language comprehension are strongly and positively associated to functional skills in the domains of social functioning (e.g., active participation at school), self-care (e.g., the child provides reliable feedback on his/her health status), and mobility (e.g., independent driving of electric wheelchair). This knowledge is very relevant for the timely planning of interventions to support communication and learning.

The study has both strength and limitations. Although the number of subjects is relatively small, SMA1 is an extremely rare condition and there are relevant difficulties to handle its most severe subtypes. The adaptation of standardized tests, by using scanning (and not eye tracker or other devices that require previous training [37]) to enable answering was a strength of the study because it allowed testing the mostly compromised children, with no necessity of motor or speech interaction [23, 25, 29, 35]. However, testing took a lot of time and children needed many pauses, making hard to perform the whole assessment all in 1 day, so that only children with higher attention spans were able to complete both tests in the same session. Probably the two tests should be administrated in two different sessions to avoid dropouts.

The absence of a control group could be a limitation; however, this should also be considered a feasibility study on the possibility to assess cognitive abilities and language comprehension in routine clinical practice. Widely introducing appropriate test adaptation to motor and language limitations is essential to prevent the risk of underestimating cognitive competences and language comprehension in children with complex disabilities. It would allow a more complete and accurate assessment of the potential of the child and his/her family, thereby ensuring more efficient and family-centered treatment plans. The relationships between the child’s functioning in his/her cognitive, communicative, and the level of parental stress should also be routinely evaluated and studied. We decided to better focus on this topic in further papers, as it is a large and interesting feature, which characterizes the whole life context of a family with a SMA child, being also part of our routine assessment. Further studies will also focus on augmentative and alternative communication intervention and its outcome in this sample, according to the results of future retrospective and/or follow-up evaluations.

Considering clinically homogeneous SMA1 natural history patients was another strength. This could represent a benchmark to evaluate the effect of new therapeutic options, either already commercially available or under approval [14, 36–41]. Being a single-center retrospective study was a weakness of this study and further prospective multicenter studies are needed to systematically collect data on the linguistic and intellectual development of children with SMA1, with particular attention to disease severity which has also been shown to play a role in the response to treatment [42].

## Conclusions

Our results provided the first knowledge on speech intelligibility, cognitive development, and morphosyntactic comprehension in SMA1 children, and on feasible routine strategies to adapt testing to severe motor and speech limitation. Despite deprivation of normal developmental stimulation and of very severe limitations in motor function, it appears that intellectual skills and internal language, and particularly morphosyntactic comprehension, are not affected in SMA1 children whereas quality of speech is severely affected and dependent on disease severity.

## Data Availability

Single data are presented in Table 1 and in Figs. 2 and 3.

## Abbreviations

ALS: Amyotrophic lateral sclerosis
ALSSS: ALS Severity Score
CHOP-Intend: Children’s Hospital of Philadelphia Infant Test of Neuromuscular Disorders
IQ: Intelligence quotient
SMA: Spinal muscular atrophy
SMA1: Spinal muscular atrophy type 1
SMN: Survival of motor neuron
RCPM: One-dimensional Raven test
TCGB: Test di Comprensione Grammaticale del Bambino (Brown Bellugy modified for Italian standards)
